# Case report: Coexistence of Labbe vein thrombosis and autoimmune encephalitis with two different antibodies

**DOI:** 10.3389/fneur.2023.1170169

**Published:** 2023-07-13

**Authors:** Lu Yang, Dongqing Zhang

**Affiliations:** Department of Pediatrics, Qilu Hospital, Shandong University, Jinan, Shandong, China

**Keywords:** venous thrombosis, autoimmune encephalitis (AE), child, MRI, NMDAR, GABABR

## Abstract

Anti-NMDA receptor encephalitis is an autoimmune encephalitis well- known to pediatric neurologists. The characteristic combination of symptoms and detection of NMDA receptor antibody can confirm the diagnosis. Most children respond well to immunosuppressive therapy. Anti-GABAB receptor encephalitis usually occurs in adult patients. Most patients present clinically with symptoms of limbic encephalitis. Cases in pediatric patients are rare. Cerebral venous thrombosis also has a very low incidence in children without underlying diseases. Patients usually present with headaches, convulsions, and focal deficits. Anticoagulants are the first choice treatment. We report a boy initially diagnosed with Labbe vein thrombosis and later tested positive for both NMDA and GABAB receptors. Anticoagulants did not relieve the boy's symptoms, and immunosuppressive therapy achieved good results. The antibody titers were significantly reduced or even turned negative. Although the Labbe vein was not recanalized at four months follow-up, the brain lesion was significantly absorbed. We learn from this case that a child can be inflicted with cerebral venous thrombosis and autoimmune encephalitis simultaneously. Child patients respond well to treatment.

## Introduction

Anti-NMDA receptor encephalitis is one of the most commonly diagnosed autoimmune encephalitis among children. Children usually present symptoms including acute behavioral change, seizures, language dysfunction, and prominent dyskinesias after prodromal headache, fever, or a viral-like process (1). Brain MRI can be normal or nonspecific changes in cortical or subcortical regions (2). An ovarian teratoma might be detected in older girl patients (1). Anti-GABAB receptor encephalitis is primarily encountered in adult patients and often associates with small-cell lung cancer (3). The main symptoms are similar to those of anti-NMDA receptor encephalitis, including cognitive deficit, behavior disorders, impairment of consciousness, movement disorder, seizure, etc. (4). Brain lesion usually occurs in the medial temporal lobe (4). Labbe vein thrombosis is a relatively uncommon entity. Headaches and seizures are the most commonly described clinical presentations of Labbe vein thrombosis (5). This is the first report of a child who simultaneously had Labbe vein thrombosis and tested positive for both NMDA and GABAB receptor antibodies.

## Case report

The boy was admitted for intermittent headaches for two weeks after jumping rope. Each headache attack lasted for several hours and resolved spontaneously. The headache got worse 3 days ago. The boy had normal intellectual development and no history of critical illness. On the day of admission, the boy suffered from headaches and was unwilling to communicate with others. Physical examination did not reveal other positive signs.

**Figure 1:**
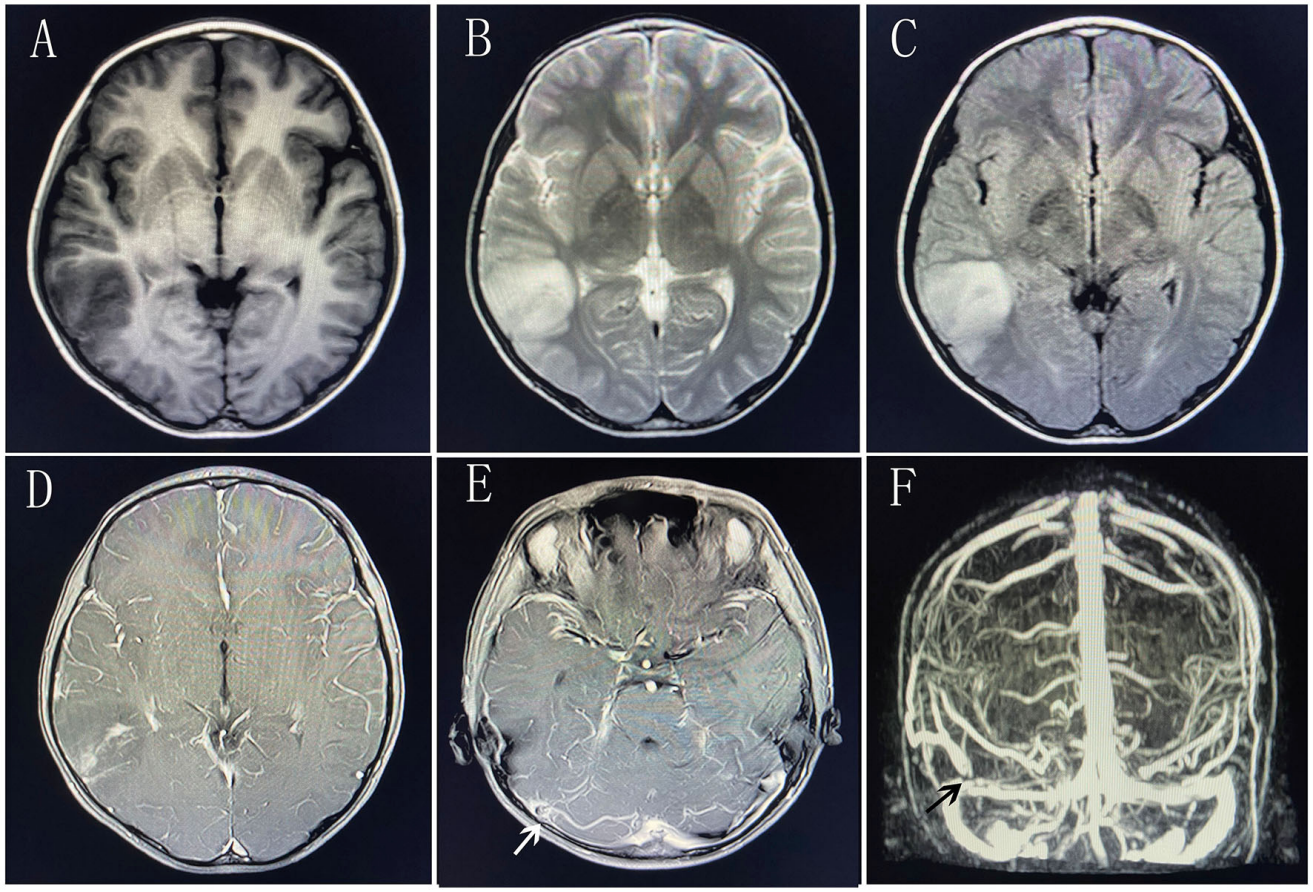
Brain magnetic resonance imaging at first administration. **(A)** T1-weighted image. **(B)** T2-weighted image. **(C)** Flair sequence. **(D)** Gadolinium-enhanced T1-weighted image of the right temporal lobe. **(E)** Gadolinium-enhanced T1-weighted image of the transverse sinus. The white arrow indicates the filling defect of right transverse sinus. **(F)** MRV scanning. The black arrow indicates the interruption of blood flow at the junction of the right Labbe vein and right transverse sinus.

An initial brain magnetic resonance imaging (MRI) revealed hyperintensity in the right temporal lobe on T2 and fluid attenuated inversion recovery (Flair) sequences. Gadolinium contrast MRI scanning revealed patchy and strip-like enhancement in this area. MR angiography showed no abnormality in the arterial system. MR venography showed the relatively thickened distal part of the right Labbe vein and stenosis at the junction of the right Labbe vein and right transverse sinus, suggesting of thrombosis. Gadolinium-enhanced T1-weighted image further confirmed the clogging in the vein (Figure 1). Extensive blood tests were all normal, including a complete blood count, blood chemistry, coagulation function, anticoagulants concentration, rheumatic disease antibodies, and vasculitis antibodies (Table 1). Cerebrospinal fluid (CSF) tests revealed normal intracranial pressure (165 mmH_2_O), leucocytosis (90 cells/mm^3^), normal glucose level, slightly increased immunoglobulin (IgG 39.50 mg/L, IgA 5.72 mg/L, IgM 18.2 mg/L) and negative pathogen finding in next generation sequencing.

**Table 1:** Blood test results.

**Blood test**	**Result**	**Reference value**
Leucocyte ( × 10^9^/L)	4.98	3.5–9.5
Hemoglobulin (g/L)	143	110–140
Platelet ( × 10^9^/L)	254	125–350
Lactic acid (mmol/L)	1.9	0.7–2.1
Ammonia (umol/L)	30	9–33
Antithrombin III (%)	118	80–120
Fibrinogen (g/L)	3.57	2.00–4.00
D-dimer (ug/ml)	0.41	< 0.5
ALT (U/L)	9	9–50
Creatine (umol/L)	46	27–53
PR3-ANCA (U/ml)	1.42	< 5
MPO-ANCA (U/ml)	1.77	< 5
ACL-IgG (U/ml)	0.82	< 10
ACL-IgM (U/ml)	9.23	< 10
ANA	Negative	Negative
dsDNA (IU/ml)	< 10.0	< 100.0
RF (IU/ml)	< 11.3	< 15

**Figure 2:**
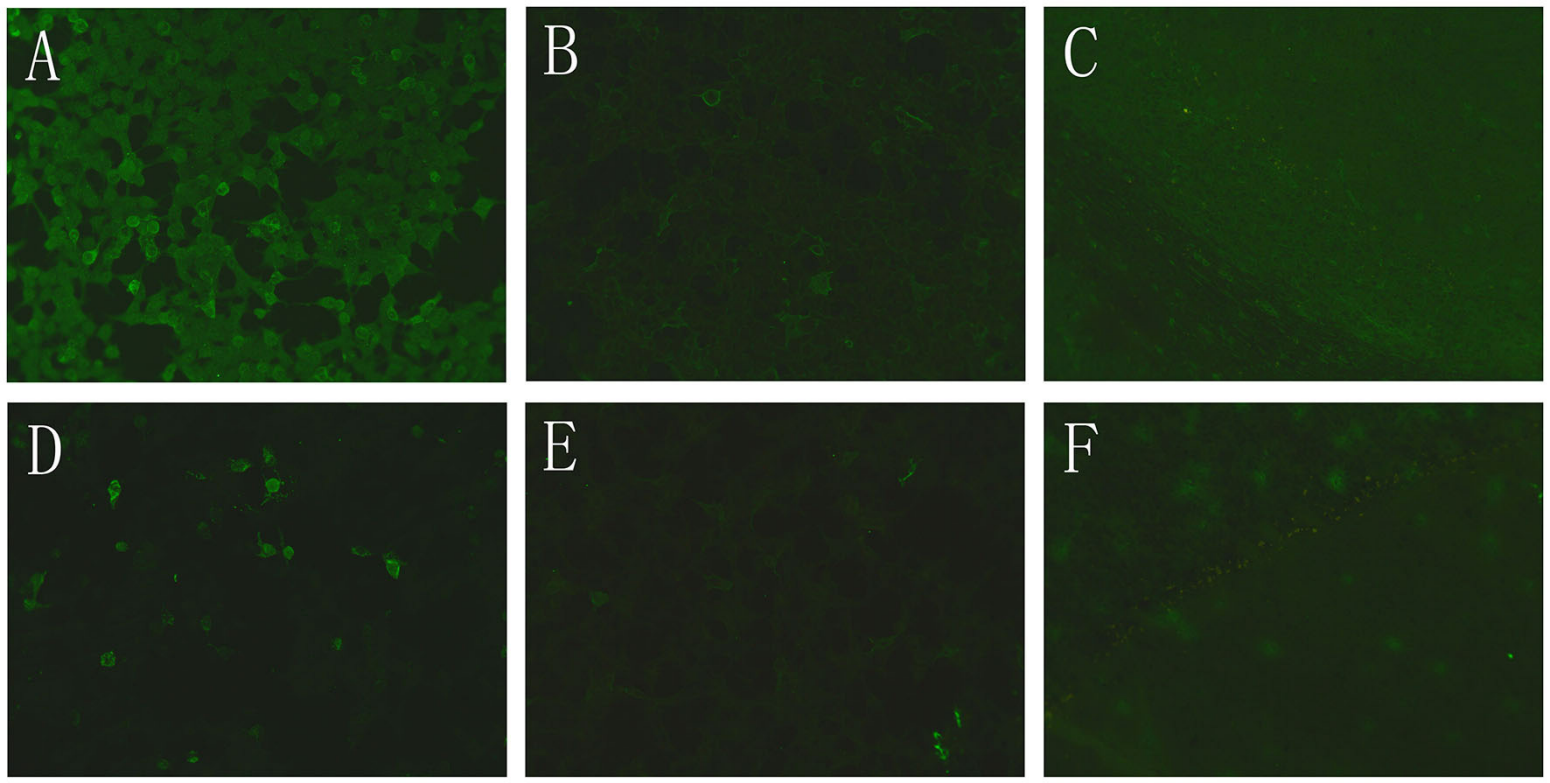
Indirect immunofluorescence assay of autoimmune encephalitis antibodies. **(A)** Cell based assay of NMDAR antibody in blood (1:10 titer). **(B)** Cell based assay of GABABR antibody in blood (1:10 titer). **(C)** Tissue based assay for blood antibodies against monkey cerebellum. **(D)** Cell based assay of NMDAR antibody in CSF (1:10 titer). **(E)** Cell based assay of GABABR antibody in CSF (1:1 titer). **(F)** Tissue based assay for CSF antibodies against monkey cerebellum.

**Figure 3:**
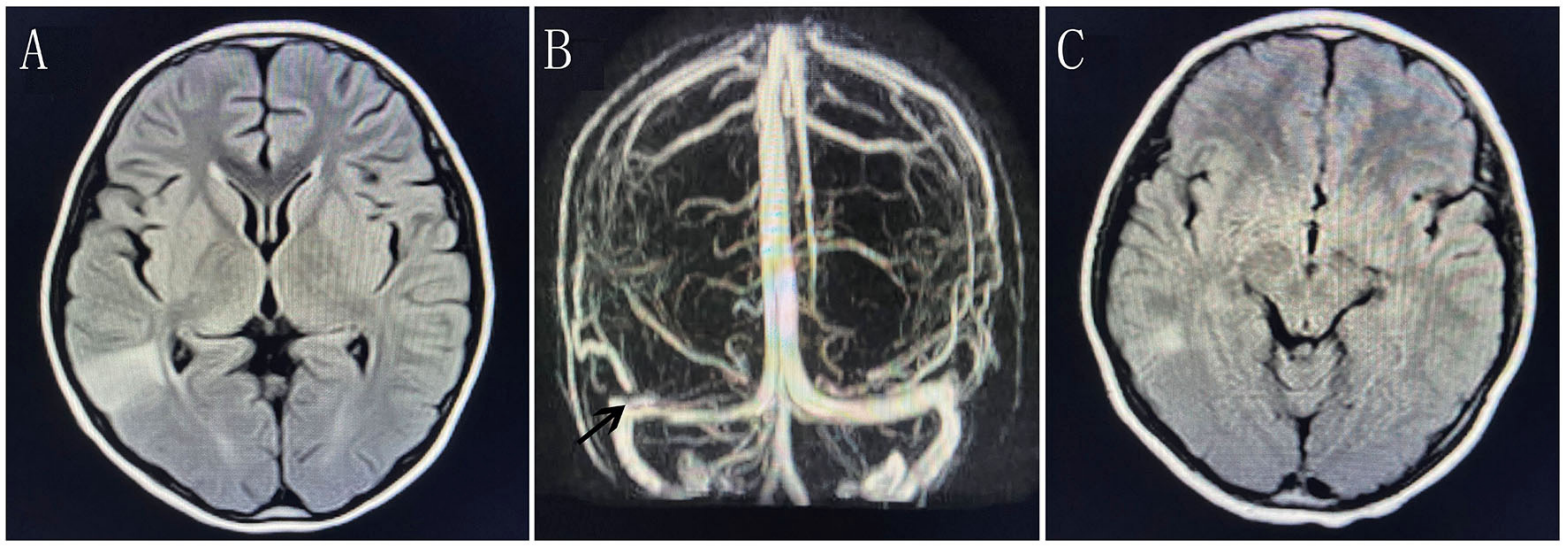
Brain magnetic resonance imaging at follow-up. **(A)** Flair sequence before discharge. **(B)** MRV scanning at four-month follow-up. The black arrow indicates the interruption of blood flow at the junction of the right Labbe vein and right transverse sinus. **(C)** Flair sequence at four-month follow-up.

Before the autoimmune encephalitis antibody results returned, the boy was given immunoglobulin (2 g/kg) and anticoagulant therapy with low molecular weight heparin. However, the boy's symptoms aggravated with paroxysmal irritability, crying, and intermittent complaint of abdominal pain. The boy demonstrated mood instability, ranging from overexcited to indifferent and uncommunicable. The boy had convulsions on the 9th and 12th hospital days. Electroencephalogram (EEG) recording showed spike and slow waves in the right temporal and occipital region. At this time, autoimmune encephalitis antibody analysis showed that both NMDA receptor antibody and GABAB receptor antibodywere positive in the blood (NMDAR receptor antibody titer 1:10, GABAB receptor antibody titer 1:10) and CSF (NMDA receptor antibody titer 1:10, GABAB receptor antibody titer 1:1) (Figure 2). Autoimmune encephalitis was diagnosed. No evidence of tumor was found on chest, abdomen, and pelvis computed tomography (CT) scanning. The boy was administered large doses of methylprednisolone (20 mg/kg/d) 3 days a week for two weeks, followed by two dosages of rituximab (375 mg/m^2^/week). The boy's condition improved significantly, with a stable mood and normal verbal communication. He no longer had headaches and convulsions. The right temporal lobe lesion was partially absorbed on a follow-up brain MRI scanning before discharge (Figure 3A). After discharge, the boy took oral corticosteroid and rivaroxaban. Four months later, the boy fully recovered without any neurological deficit. Repeated analysis for autoimmune encephalitis antibodies showed the NMDA receptor antibody titer in CSF dropped to 1:1, and the GABAB receptor antibody was negative. No significant CSF leucocytosis (4 cells/mm^3^) was present. The stenosis at the junction of the right Labbe vein and transverse sinus remained unchanged and the right temporal lobe lesion nearly completely disappeared (Figures 3B, C).

## Discussion

Cerebral venous thrombosis (CVT) may present with highly variable symptoms. The onset can be acute, subacute, or chronic (6). CVT mostly presents with headache, which is observed in more than 3/4 of the patients (7). Additional symptoms include focal neurological deficits, seizures, and encephalopathy (7). The patients with CVT often have high D-dimer levels and increased intracranial pressure. A normal D-dimer level could not preclude venous thrombosis because patients with longer duration of symptoms and lesser clot burden could have normal D-dimer (8). Not all CVT survivors had intracranial hypertension in a 293-patients study (9). Age, sex-specific factors, and hereditary thrombophilia were independently associated with intracranial hypertension (9). The Labbe vein is part of the superficial venous system, which connects the superficial middle cerebral vein and the transverse sinus. It arises in the Sylvian fissure and travels posteriorly and inferiorly into the transverse sinus. The Labbe vein territory is defined as the lateral surface of the temporal lobe and does not include the medial part of the temporal lobe. The boy's brain lesion on MRI scanning was completely consistent with the Labbe vein territory. Although this boy's intracranial pressure was within the normal range and the D-dimer concentration did not increase, his MRV image and Gadolinium-enhanced image confirmed the presence of a thrombus at the junction of right Labbe vein and transverse sinus. Our boy's thrombophilia screening and rheumatoid screening were all negative. As a mechanical precipitant, jumping the rope might be the only inducer of the thrombosis.

The encephalitis antibody panel revealed the presence of NMDA and GABAB receptor antibodies in the blood and CSF. CSF pleocytosis in our boy patient is consistent with the characteristic of autoimmune encephalitis. Immunotherapy with immunoglobulin, large dose methylprednisolone, and rituximab significantly relieved the boy's symptoms, which further confirmed the autoimmune encephalitis diagnosis. There were many reports of the coexistence of NMDA receptor antibody and other antibodies, such as MOG antibody, GAD65 antibody, Caspr2 antibody, and mGluR5 antibody (10–12). This is the first report of the coexistence of NMDA receptor antibody and GABAB receptor antibody, which might be explained by the coexpression of NMDA receptors and GABAB receptors on neuron membranes.

Some systemic autoimmune diseases can lead to cerebral venous sinus thrombosis, such as Behcet's syndrome, systemic lupus erythematosus, antiphospholipid syndrome, and Sjogren's syndrome (13). Vasculitis and endothelial cell impairment caused by autoantibodies are the pathological mechanisms of thrombosis in some of these systemic diseases. There were also reports of comorbidity of anti-NMDAR encephalitis and cerebral venous thrombosis (14, 15). Neither the NMDA receptor antibody nor the GABAB receptor antibody could cause vasculitis. So we propose the brain tissue damage caused by venous thrombosis triggered immune response and produced the two different autoimmune antibodies.

According to previous reports, most of the patients with anti-NMDA receptor encephalitis (16) or anti-GABAB receptor encephalitis (3) achieved good outcomes after treatment. In our case, immunosuppressive therapy also significantly relieved the boy's symptoms in the acute stage.

Meta-analysis revealed that 15% of patients with cerebral venous thrombosis could not achieve recanalization. Functional recovery could be achieved in 71% of patients without recanalization (17). The right Labbe vein of our boy was not recanalized at 4 months follow-up. However, the right temporal lobe lesion shrank significantly.

## Conclusion

In this case, the boy's poor response to anticoagulants warrants further analysis for autoimmune encephalitis panel. Although the causal relationship between Labbe vein thrombosis and autoimmune encephalitis can not be determined, we know that patients can be inflicted with these two diseases simultaneously. NMDA receptor antibody and GABAB receptor antibody can also coexist in one patient. Favorable outcomes can be achieved in this complex clinical condition in child patients.

## Data availability statement

The original contributions presented in the study are included in the article/supplementary material, further inquiries can be directed to the corresponding author.

## Ethics statement

The studies involving human participants were reviewed and approved by the Ethics committee on scientific research of Shandong University Qilu Hospital. Written informed consent to participate in this study was provided by the participants' legal guardian/next of kin. Written informed consent was obtained from the minor (s)' legal guardian/next of kin for the publication of any potentially identifiable images or data included in this article.

## Author contributions

LY prepared the clinical data and drafted the manuscript. DZ supervised this case report. All authors analyzed and interpreted the patient data and revised the manuscript. All authors contributed to the article and approved the submitted version.
